# Asymmetric expression of Argonautes in reproductive tissues

**DOI:** 10.1093/plphys/kiab474

**Published:** 2021-10-23

**Authors:** P E Jullien, J A Schröder, D M V Bonnet, N Pumplin, O Voinnet

**Affiliations:** 1 Institute of Plant Sciences, University of Bern, 3012 Bern, Switzerland; 2 Institute of Molecular Plant Biology—Swiss Federal Institute of Technology Zurich (ETH‐Zurich), 8092 Zurich, Switzerland

## Abstract

The Arabidopsis genome encodes ten Argonautes proteins showing distinct expression pattern as well as intracellular localisation during sexual reproduction.

Dear Editor,

Small RNAs (sRNAs) are key regulators of gene expression. The importance of reproduction-specific sRNAs has been recognized in plants (Mosher and Melnyk, 2010; Van Ex et al., 2011). Beyond the roles of microRNAs (miRNAs) in embryonic development, small interfering (si)RNAs tame transposons in pollen (Calarco et al., 2012) and during ovule development (Olmedo-Monfil et al., 2010). siRNAs are linked to hybrid seed lethality as suggested by their implication in regulating parental genome dosage (Borges et al., 2018; Martinez et al., 2018). RNA silencing in plants can be divided into Post-Transcriptional Gene Silencing (PTGS) and Transcriptional Gene Silencing (TGS) processes. Both pathways rely on the generation of sRNAs of 21, 22, or 24 nucleotides in length by DICER-LIKE enzymes. These sRNAs execute PTGS or TGS upon their loading into ARGONAUTE (AGO) effector proteins. Despite the established impact of sRNA-pathway mutations in plant biology, little is known of the expression profiles of silencing-pathway proteins in reproductive tissues. To fill this gap, we have generated stable transgenic lines expressing full length fluorescently tagged AGOs in N-terminal under their cognate endogenous promoter and analyzed their expression as well as intracellular localization in reproductive tissues (Supplemental Figures S1–S3).

## AGO expression patterns in mature gametophytes

**Figure 1:**
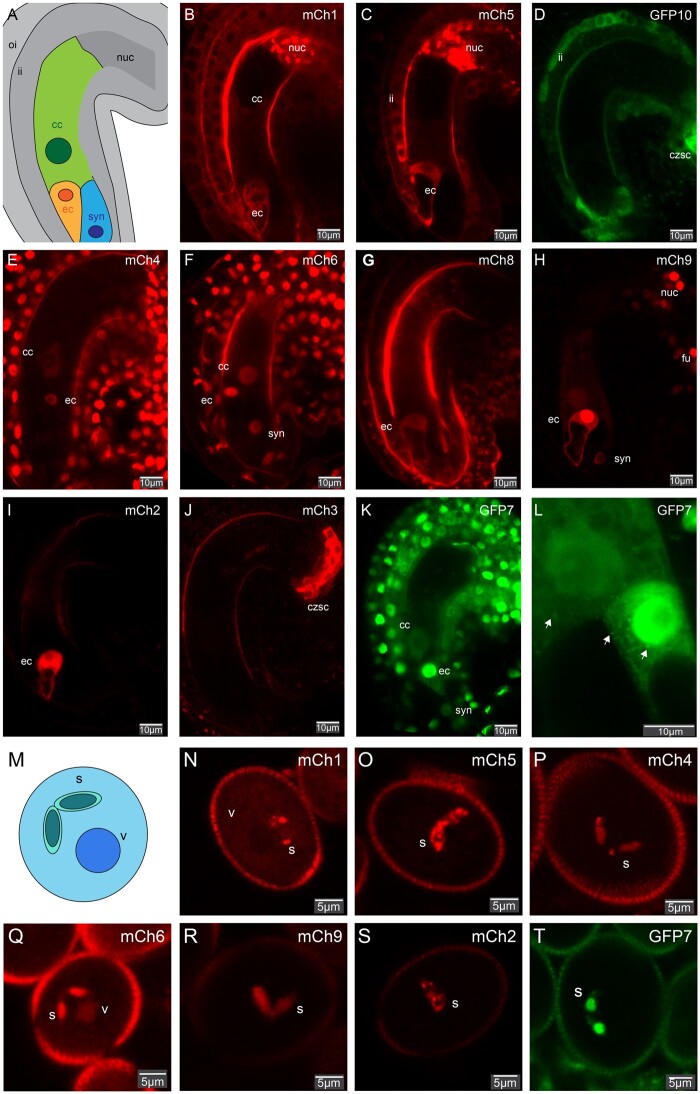
AGOs accumulation in the mature female and male gametophytes. A, Schematic representation of a mature female gametophyte of *Arabidopsis thaliana* illustrating the three major cell types: the central cell (cc) in green, the egg cell (ec) in orange, the synergides (syn) in blue. The mature female gametophyte is surrounded by maternal sporophytic tissue represented in gray, including the inner integument (ii), outer integument (oi), and the nucellus (nuc). B–K, Confocal images representing the expression of the 10 *Arabidopsis* AGOs in mature female gametophytes: mCherry-AGO1 (B), mCherry-AGO5 (C), GFP-AGO10 (D), mCherry-AGO4 (E), mCherry-AGO6 (F), mCherry-AGO8 (G), mCherry-AGO9 (H), mCherry-AGO2 (I), mCherry-AGO3 (J), and GFP-AGO7 (K). L, Confocal image of the intra-cellular localization of GFP-AGO7 in the egg cell and central cell. Scale bars represent 10 µm. Additional channels and pictures can be found in Supplemental Figures S4 and S5. M, Schematic representation of a mature pollen grain of *A. thaliana* illustrating the two major cell types: the vegetative cell (v) and the two sperm cells (s). N–T, Confocal images representing the expression of seven *Arabidopsis* AGOs accumulating in the pollen grain: mCherry-AGO1 (N), mCherry-AGO5 (O), mCherry-AGO4 (P), mCherry-AGO6 (Q), mCherry-AGO9 (R), mCherry-AGO2 (S), and GFP-AGO7 (T). Additional pictures including DAPI staining can be found in Supplemental Figure S7. Scale bars represent 5 µm. mCherry-AGO1, mCherry-AGO5, and mCherry-AGO2 cytosolic aggregates observed in the nucellus and sperm cells (panels B, C, N, O, and S) are likely due to artifactual aggregation, as previously reported with mCherry-tagged proteins (Landgraf et al., 2012; Cranfill et al., 2016) and as they occur only in tissues where AGO1, AGO5, and AGO2 are particularly highly expressed.

Almost all AGOs accumulate in the mature female gametophyte except GFP-AGO10 and mCherry-AGO3 (Figure  1, A–K and Supplemental Figure S4). GFP-AGO10 mainly accumulates in the inner-integument of the ovule with a stronger signal at the chalazal seed coat and in vascular tissues of the funiculus (Figure  1, D and Supplemental Figure S5, B) while mCherry-AGO3 is solely detected in the chalazal seed coat of the ovule (Figure 1, J;Jullien et al., 2020). All other AGOs accumulate preferentially in the egg cell. While mCherry-AGO1/5/4/6/9 and GFP-AGO7 are also detected to a low level in the central cell; mCherry-AGO2 and mCherry-AGO8 are specific of the egg cell (Figure  1 and Supplemental Figure S5, A). Some AGOs also accumulate in the maternal integument, with mCherry-AGO1/4/6 and GFP-AGO7 being detected in both the inner and outer integument, while mCherry-AGO5 and GFP-AGO10 solely in the former, as previously reported for AGO5 (Tucker et al., 2012). Additionally, several AGOs display a strong expression in the nucellus at the chalazal pole of the ovule, particularly, mCherry-AGO1, mCherry-AGO5, and mCherry-AGO9. Strong mCherry-AGO9 accumulation is also detected in the funiculus (Figure  1, H and Supplemental Figure S5, C), consistent with previous in situ hybridization results (Olmedo-Monfil et al., 2010). Egg cell expression of both AGO8 and AGO9 is supported by transcriptional fusions (Sprunck et al., 2019). However, mCherry-AGO9 expression in the egg cell does not agree with previous immuno-localization (Olmedo-Monfil et al., 2010). Overall, our analysis shows that all 10 *Arabidopsis* AGOs are detected in mature ovules before fertilization. Within the female gametophyte, their accumulation seems to be particularly enriched in the egg cell compared with the central cell. Preferential AGO expression in the egg cell was confirmed using previously published female gametophyte transcriptome data obtained by laser-capture microdissection (Supplemental Figure S6; Wuest et al., 2010).

Analysis of the translational reporters in mature pollen grains (Figure  1, M–T and Supplemental Figure S7) revealed a preferential enrichment of some AGOs in sperm cells. Indeed, mCherry-AGO2/4/9 and GFP-AGO7 were solely detected in those cells. mCherry-AGO1/6 were mainly detected in sperm cells, but also in the vegetative cell at a lower level. AGO5 is expressed in sperm cells as previously reported (Borges et al., 2011; Tucker et al., 2012). Signals from the mCherry-AGO3, mCherry-AGO8, and GFP-AGO10 reporters were not detected in mature pollen. To address if AGOs could potentially be transmitted during fertilization, we investigated the presence of the fusion proteins’ fluorescent signals in germinated pollen (Supplemental Figure S8). All AGOs exhibiting sperm cells expression were also detected in germinated pollen. Additionally, to know if paternal expression of those AGOs could be detected in the early embryo, we analyzed AGO’s paternal expression in zygotes at 17 h-after-pollination (17HAP) (Supplemental Figure S9). Despite their very low level, we could detect paternal AGO expression for all tested AGOs in the 1-cell zygote. These results suggest that AGO-loaded sRNAs of paternal origin could be transported to the egg cell and potentially regulate gene expression in the zygote at, or shortly after, fertilization. However, considering the high egg cell AGO expression and the low AGO paternal expression in the early zygote, a strong maternal bias is expected in the early zygote.

## AGO expression patterns after fertilization

**Figure 2:**
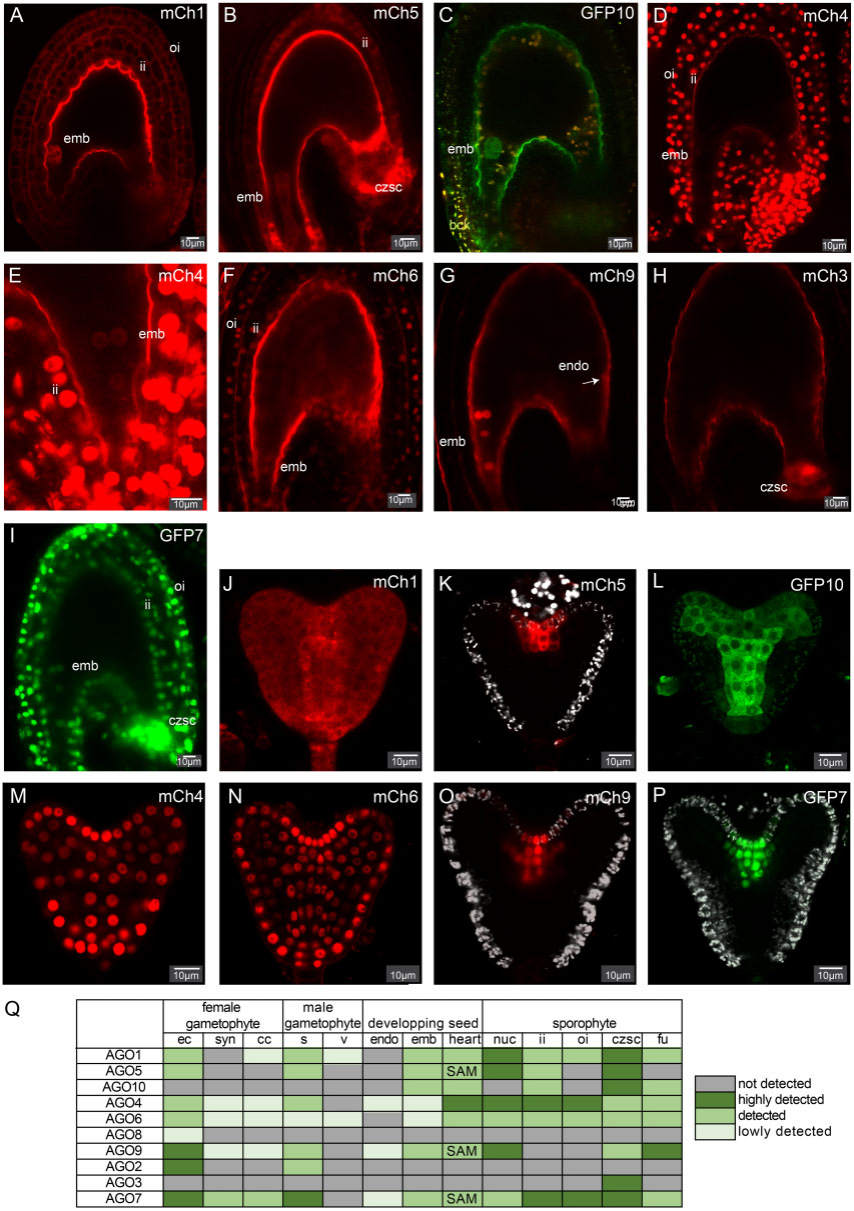
AGOs accumulation in the developing seed. A–I, Confocal images of the eight *Arabidopsis* AGOs expressed in the developing seeds 2DAP: mCherry-AGO1 (A), mCherry-AGO5 (B), GFP-AGO10 (C), mCherry-AGO4 (D and E), mCherry-AGO6 (F), mCherry-AGO9 (G), mCherry-AGO3 (H), and GFP-AGO7 (I). The background (bck) fluorescence is represented in yellow in panel C. Additional channels and pictures can be found in Supplemental Figure S10. J–P, Confocal images of the seven Arabidopsis AGOs accumulating in the heart-stage embryo: mCherry-AGO1 (J), mCherry-AGO5 (K), GFP-AGO10 (L), mCherry-AGO4 (M), mCherry-AGO6 (N), mCherry-AGO9 (O), and GFP-AGO7 (P). The background (bck) fluorescence is represented in gray scale in panels K, O, and P. Scale bars represent 10 µm. Q, Summary table of AGO’s expression pattern in reproductive tissues. Abbreviation: emb (embryo 1DAP), endo (endosperm 1DAP), inner integument (ii), outer integuments (oi), Chalazal seed coat (czsc), nucellus (nuc), funiculus (fu), egg cell (ec), Synergides (syn), central cell (cc), sperm cells (s), vegetative cell (v), and heart stage embryo (heart).

At 2 day-after-pollination (2 DAP), we could detect expression of 8 out of 10 *Arabidopsis* AGOs, while signals from mCherry-AGO2 and mCherry-AGO8 are below detection limit (Figure  2, A–I and Supplemental Figure S10). mCherry-AGO3 is solely expressed in the chalazal integuments. All remaining AGOs are detected in the 2 DAP embryo but not the 2 DAP endosperm except mCherry-AGO9. mCherry-AGO9 is visible from the first nuclear division of the endosperm, upon which its signal decreases in intensity, although it is still detected in the endosperm at the 4-cells embryo stage (Supplemental Figure S5, C). GFP-AGO7 and mCherry-AGO4 could be detected in early endosperm at 17HAP but not at 2DAP (Figure  2 and Supplemental Figure S5, D and E). Based on our reporter constructs, AGO9 appears therefore, to be the only AGO detectable in the endosperm at 2DAP. However, unlike our observation, AGO1 and AGO4 transcripts are detected in the late endosperm (3DAP) (Supplemental Figure S11). This discrepancy could be due to the stage or regulation at the protein level. Like in ovules before fertilization, mCherry-AGO1/4/6 and GFP-AGO7 are detected in both inner and outer integument while mCherry-AGO5 and GFP-AGO10 only in the former. To conclude, we observe a strong asymmetry of AGOs’ patterns between the endosperm and the embryonic lineages. This difference is supported by LCM transcriptomic data from developing seeds (Supplemental Figure S11; Belmonte et al., 2013) and suggests a less active involvement of RNA silencing pathways in the endosperm compared with the embryo, during early seed development.

We then analyzed AGO accumulation patterns in the differentiated zygote at the heart-stage (Figure  2, J–P) As previously reported (Lynn et al., 1999; Du et al., 2019), mCherry-AGO1 is expressed in all cells of the heart-stage embryo. Similarly, we observed a ubiquitous expression of mCherry-AGO4 and mCherry-AGO6. By contrast, mCherry-AGO2/3/8 could not be detected in the heart-stage embryo. GFP-AGO10 display its previously reported pattern (Tucker et al., 2008; Du et al., 2019), with fluorescent signals observed in the adaxial part of cotyledons and in the pre-vasculature. mCherry-AGO5/9 and GFP-AGO7 accumulate specifically in the shoot apical meristem (SAM) region of the heart-stage embryo, a pattern already documented for AGO5 (Tucker et al., 2012). In agreement with our results, AGO5, AGO7, and AGO9 transcript were found to be enriched in meristematic stem cells (Gutzat et al., 2020). Although AGO7 and AGO9 functions in meristems remain to be investigated, recent work suggests that AGO5 is involved in regulating flowering, perhaps via a floral meristem-specific activity (Roussin-Léveillée et al., 2020).

## AGO intracellular localization patterns

*Arabidopsis* AGO1 and AGO4 have been shown to shuttle between the cytoplasm and the nucleus although their respective steady-state subcellular localizations seem to reflect their involvement in either PTGS (AGO1, cytoplasmic) or TGS (AGO4, nuclear) (Ye et al., 2012; Bologna et al., 2018). In agreement, mCherry-AGO1/5/2/3 and GFP-AGO10 are mostly localized in the cytoplasm in reproductive cells (Figure  1, B–D, I and J) while the main TGS AGOs, mCherry-AGO4 and mCherry-AGO6, are localized in the nucleus in all reproductive cell types analyzed as previously observed in other tissues (Zheng et al., 2007; Ye et al., 2012). Perhaps more strikingly, mCherry-AGO9 displays nuclear localization in somatic tissues but appears to be also partially localized to the cytoplasm in the central and egg cells (Figure  1, H). Combined cytoplasmic and nuclear localizations of AGO9 were previously observed by immunolocalization in ovule primordia (Rodríguez-Leal et al., 2015; Zhao et al., 2018). The mCherry-AGO8 translational reporter, unlike other TGS AGOs, was mainly localized to the cytoplasm of the egg cell (Figure  1, G) likely because AGO8 is a truncated protein (Takeda et al., 2008). One of the most intriguing intracellular localization patterns was that of GFP-AGO7, found mainly localized to the nucleus. However, in some cells of the integument, the egg cell and the central cell, clear cytosolic localization was additionally observed, with the presence of cytoplasmic foci (Figure  1, L). Localization of GFP-AGO7 to cytoplasmic foci named “sRNA bodies” was previously observed (Jouannet et al., 2012) but nuclear GFP-AGO7 accumulation was not reported in this study.

To conclude, our study reveals a clear asymmetry of AGO expression between the gametes and accessory cells before fertilization and between the embryo and endosperm lineage after fertilization. A summary of their expression pattern in reproductive tissue can be found in Figure  2, Q. Fluorescent AGO reporters have been sporadically described in *Arabidopsis*, yet they are often marred by biological incongruities such as the use of overexpression promoters or C-terminal fusions known to affect AGO functions. The uniform set of tools presented in this study might help bridging this gap across the plant RNA silencing community.

## Supplemental data

**Supplemental Figure S1.** Schematic representation of the constructs used in this study.

**Supplemental Figure S2.** RT-qPCR results assessing AGOs expression levels in inflorescence of wild-type and complemented lines.

**Supplemental Figure S3.** Complementation of ago mutants.

**Supplemental Figure S4.** Additional channels and LUTs corresponding to the pictures of Figure  1.

**Supplemental Figure S5.** Additional pictures.

**Supplemental Figure S6.** *Arabidopsis* AGO transcription patterns extracted from microarray data of LCM-dissected female gametophytes (Wuest et al., 2010) confirming the general enrichment of AGO transcripts in the egg cell (EC) compared with central cell (CC) or synergids (Syn).

**Supplemental Figure S7.** Additional pictures of AGOs expression pattern in mature pollen counter stained with DAPI.

**Supplemental Figure S8.** AGO accumulation in germinating pollen tube.

**Supplemental Figure S9.** Paternal expression in the early zygote of AGOs expressed in sperm cells.

**Supplemental Figure S10.** Additional channels and LUT corresponding to the pictures of Figure  2, A–I.

**Supplemental Figure S11.** *Arabidopsis* AGO transcription patterns extracted from microarray data of LCM-dissected seeds at the pre-globular stage (Belmonte et al., 2013) confirming the general enrichment of AGO transcripts in the embryo compared with the peripheral endosperm.

**Supplemental Table S1.** Primers used in this study.

**Supplemental Text.** Materials and Methods.

## Supplementary Material

kiab474_Supplementary_Data

## Notes

P.E.J. conceived the study. P.E.J., N.P., and J.A.S. generated the transgenic lines. P.E.J., J.A.S., and D.M.V.B. performed the imaging. D.M.V.B. and J.A.S. tested the complementation. P.E.J. wrote the manuscript, which was further edited and amended by O.V.

The author responsible for distribution of materials integral to the findings presented in this article in accordance with the policy described in the Instructions for Authors (https://academic.oup.com/plphys/pages/General-Instructions) is P. E. Jullien (pauline.jullien@ips.unibe.ch).
