# Supplementary material for: Asymmetric expression of Argonautes in reproductive tissues
Source: Plant Physiol. 2021 Oct 23;188(1):38–43. doi: 10.1093/plphys/kiab474 (PMC8774725; doi:10.1093/plphys/kiab474)
Supplement: kiab474_Supplementary_Data [file kiab474_supplementary_data.pdf]

SUPPLEMENTAL TEXT

MATERIALS AND METHODS

PLANT MATERIAL AND GROWTH CONDITIONS

After three days at 4°C in the dark, seeds were germinated and grown on soil. Plants were grown under long days at 20-21°C (16h light/8h night). All plants were in Columbia (Col-0) accession. The mutants and lines described in this work correspond to the following: *ago1-36* (SALK_087076,(Baumberger and Baulcombe, 2005)), *ago2-1* (salk_003380,(Lobbes et al., 2006)), *ago3-3* (GABI-743B03,(Jullien et al., 2020)), *ago4-5* (WiscDsLox338A0, (Stroud et al., 2012)) , *ago5-1* (salk_063806, (Katiyar-Agarwal et al., 2007)), *ago6-2* (salk_031553, (Zheng et al., 2007)), *ago7-1* (salk_037458, (Vazquez et al., 2004)), *ago8-1* (salk_139894, (Takeda et al., 2008), *ago9-1* (salk_127358, (Katiyar-Agarwal et al., 2007)) and *ago10-1* (SALK_000457). The insertion lines were provided by The Nottingham *Arabidopsis* Stock Centre (NASC) (http://arabidopsis.info/). Pollen were germinated in Pollen growth medium at 21°C in the dark 5 hours to over-night (Hamamura et al., 2011).

MICROSCOPY

Fluorescence images were acquired using laser scanning confocal microscopy (Zeiss LSM780 or Leica SP5). The laser excitation and selected emissions were as followed: 488nm and 499-549nm for GFP, 561nm and 600-645 for mCherry, 405nm and 450-470 for DAPI and 488 or 561nm and LP650nm for the background fluorescence. Thickness of the Z-slice, gain and laser power were adjusted depending on the strength of the signal and tissue type. Brightness adjustment, LUTs, crops, stacks and merges were performed using ImageJ, Fiji (http://rsbweb.nih.gov/ij/, (Schindelin et al., 2012)) and assembled using ImageJ or Adobe Illustrator.

PLASMID CONSTRUCTION AND TRANSFORMATION

All DNA fragments were amplified by PCR using the Phusion High-Fidelity DNA Polymerase (Thermo). Primer sequences can be found in Supplementary Table S1. We generated full-locus reporter constructs in which the open reading frames (ORFs) of fluorescent proteins were fused to each AGO coding sequence in their genomic contexts, using multiple Gateway^{TM} cloning. We engineered N-terminal translational fusions, since N-terminal, but not C-terminal, tagging

preserves *Arabidopsis* AGOs' functionality (Carbonell et al., 2012). Each AGO construct was cloned under the corresponding presumptive promoter (1.3kb to 2.5kb upstream of start codons) and terminators (467bp to 1kb downstream of stop codons). This generated pAGO:FP-AGO constructs, where "FP" corresponds to either the Green Fluorescent Protein (GFP) or mCherry (mCh). Following PCR amplification, the promoters were recombined in pDONR-1-4, the FPs in pDONR1-2, the CDS and 3'end in pDONR-2-3 and finally assembled together in the pB7m34GW,0 destination vector (Karimi et al., 2005). For the sake of simplification, the constructs will be referred to FP-AGOX (where X is the number of each AGO1-10) in the main text and FPX in the figures. For example, *pAGO1:mCherry-AGO1* will be shortened to mCherry-AGO1 in the main text and to mCh1 in figures. A detail map of the constructs can be found in Supplemental Figure S1. *A. thaliana* transformation was carried out by the floral dip method (Clough and Bent, 1998). All plasmids were transformed into Col-0 and their respective mutants and for mCherry-AGO6 also in LIG1-GFP marker line (Andreuzza et al., 2010). Six to nineteen transgenic lines (T1) were analysed and showed a consistent fluorescence expression pattern using a Leica epifluorescence microscope or a Leica SP5. One to three independent lines with single insertions, determined by segregation upon BASTA selection, were used for further detailed confocal analysis. qPCRs assessing the *AGO* transcript level in the different lines can be found in Supplemental Figure S2. Although the expression of most AGOs is within the wild-type range, some are slightly under-expressed which could be due to the length of the chosen promoter or the stability of the transgenic RNA. The construct *mCherry-AGO9* show significant less expression than the wild-type *AGO9* (t-test, p=0.0003) (Supplemental Figure S2), likely due to a progressive silencing of the construct. Functional complementation was validated for AGO1, AGO4, AGO5 (partial complementation), AGO6, AGO7 and AGO10 (partial complementation using *AGO10* Col-0 sequences into Ler *zll-3* and *pnh-2* background) and can be found in Supplemental Figure S3. Complementation of *AGO3* and *AGO8* could not be investigated due to the absence of published mutant phenotype. Despite our attempts, we could not test for *AGO2* and *AGO9* complementation due to problem in reproducing reliably *ago2* and *ago9* mutant phenotypes.

RNA extraction and RT-qPCR

Total RNAs were extracted from leave tissues and inflorescences using Quiazol (Qiagen). DNase treatment, Reverse transcription, qPCR and its analysis were performed as previously described (Tirot and Jullien, 2021). *ACTIN2* (*AT3G18780*) expression was used to normalize

the transcript level in each sample. RT-qPCR primers can be found in Table S1. Result graphs and statistical tests were done on Rstudio (version 1.2.1335, www.rstudio.com).

SUPPLEMENTAL REFERENCES

Andreuzza S, Li J, Guitton A-E, Faure J-E, Casanova S, Park J-S, Choi Y, Chen Z, Berger F (2010) DNA LIGASE I exerts a maternal effect on seed development in Arabidopsis thaliana. Development **137**: 73–81

Baumberger N, Baulcombe DC (2005) Arabidopsis ARGONAUTE1 is an RNA Slicer that selectively recruits microRNAs and short interfering RNAs. Proc Natl Acad Sci U S A **102**: 11928–11933

Belmonte MF, Kirkbride RC, Stone SL, Pelletier JM, Bui AQ, Yeung EC, Hashimoto M, Fei J, Harada CM, Munoz MD, et al (2013) Comprehensive developmental profiles of gene activity in regions and subregions of the Arabidopsis seed. Proc Natl Acad Sci **110**: E435–E444

Carbonell A, Fahlgren N, Garcia-Ruiz H, Gilbert KB, Montgomery T a, Nguyen T, Cuperus JT, Carrington JC (2012) Functional analysis of three Arabidopsis ARGONAUTES using slicer-defective mutants. Plant Cell **24**: 3613–29

Clough SJ, Bent AF (1998) Floral dip: A simplified method for Agrobacterium-mediated transformation of *Arabidopsis thaliana*. Plant J **16**: 735–743

Hamamura Y, Saito C, Awai C, Kurihara D, Miyawaki A, Nakagawa T, Kanaoka MM, Sasaki N, Nakano A, Berger F, et al (2011) Live-cell imaging reveals the dynamics of two sperm cells during double fertilization in Arabidopsis thaliana. Curr Biol **21**: 497–502

Jullien PE, Grob S, Marchais A, Pumplin N, Chevalier C, Bonnet DM., Otto C, Schott G, Voinnet O (2020) Functional characterization of Arabidopsis ARGONAUTE 3 in reproductive tissue. Plant J 1796–1809

Karimi M, De Meyer B, Hilson P (2005) Modular cloning in plant cells. Trends Plant Sci **10**: 103–5

Katiyar-Agarwal S, Gao S, Vivian-Smith A, Jin H (2007) A novel class of bacteria-induced small RNAs in Arabidopsis. Genes Dev **21**: 3123–3134

Lobbes D, Rallapalli G, Schmidt DD, Martin C, Clarke J (2006) SERRATE: a new player on the plant microRNA scene. EMBO Rep **7**: 1052–8

Mallory AC, Hinze A, Tucker MR, Bouché N, Gasciolli V, Elmayan T, Lauressergues D, Jauvion V, Vaucheret H, Laux T (2009) Redundant and specific roles of the ARGONAUTE proteins AGO1 and ZLL in development and small RNA-directed gene silencing. PLoS Genet. doi: 10.1371/journal.pgen.1000646

Schindelin J, Arganda-Carreras I, Frise E, Kaynig V, Longair M, Pietzsch T, Preibisch S, Rueden C, Saalfeld S, Schmid B, et al (2012) Fiji: An open-source platform for biological-image analysis. Nat Methods **9**: 676–682

Stroud H, Greenberg MVC, Feng S, Bernatavichute Y V., Jacobsen SE (2012) Resource Comprehensive Analysis of Silencing Mutants Reveals Complex Regulation of the Arabidopsis Methylome. Cell **152**: 352–364

Takeda A, Iwasaki S, Watanabe T, Utsumi M, Watanabe Y (2008) The mechanism selecting the guide strand from small RNA duplexes is different among Argonaute proteins. Plant Cell Physiol **49**: 493–500

Tirot L, Jullien PE (2021) DNA METHYLTRANSFERASE 3 (MET3) is regulated by Polycomb Group complex during Arabidopsis endosperm development. bioRxiv

Vazquez F, Gasciolli V, Crété P, Vaucheret H (2004) The Nuclear dsRNA Binding Protein HYL1 Is Required for MicroRNA Accumulation and Plant Development, but Not Posttranscriptional Transgene Silencing. Curr Biol **14**: 346–351

Zheng X, Zhu J, Kapoor A, Zhu J-K (2007) Role of Arabidopsis AGO6 in siRNA accumulation, DNA methylation and transcriptional gene silencing. EMBO J **26**: 1691–1701

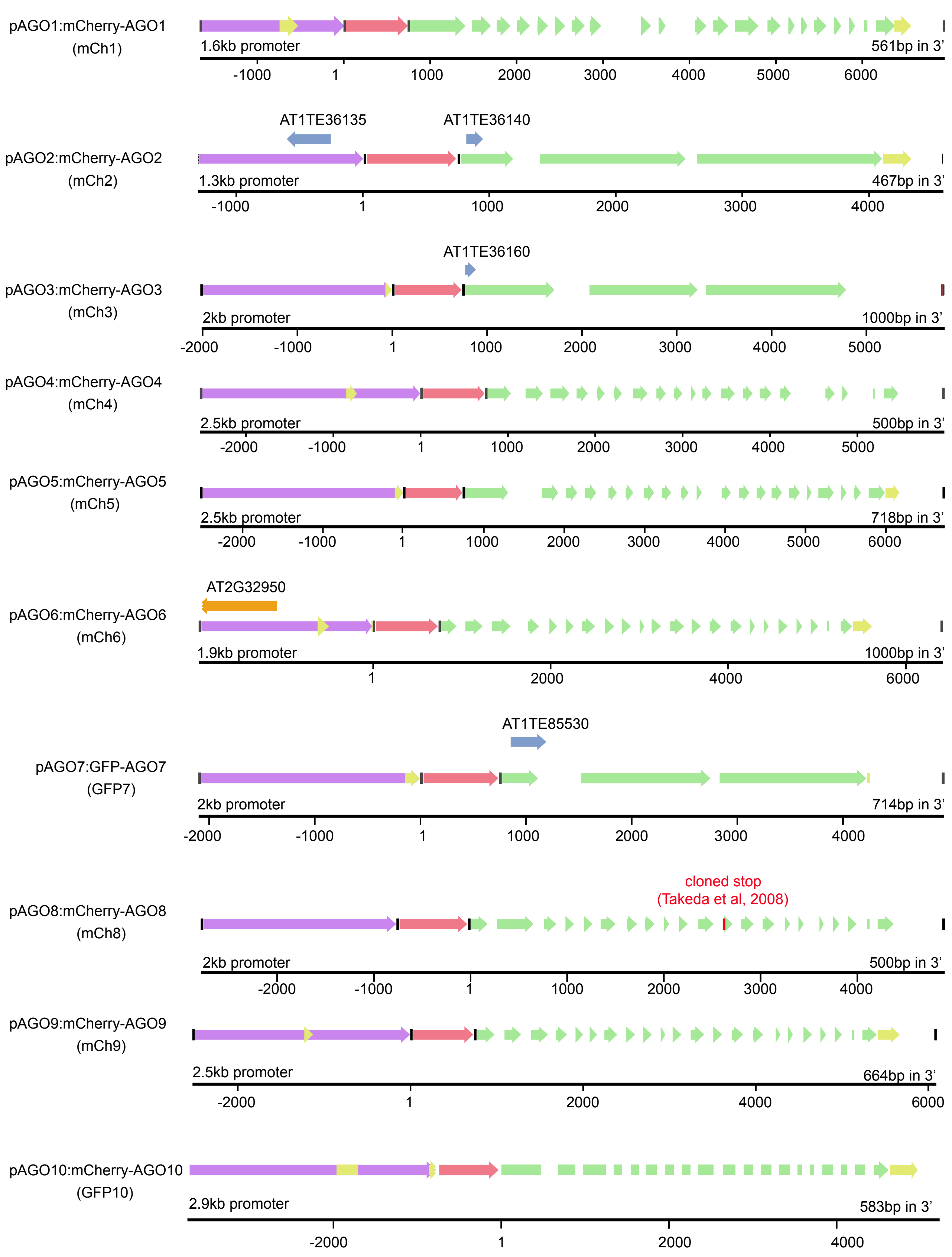

Supplemental Figure S1. Schematic representation of the constructs used in this study. The different features are represented: promoter (purple), UTRs (yellow), fluorescent protein (red), exon (green), additional TAIR10 annotations (orange) and transposable element (TE) (Blue). Promoter sequences correspond to sequences in between 2kb and 2.5kb from the ATG site except for AGO1, AGO2 and AGO10. The AGO10 promoter was extended according to Mallory et al 2009. The AGO2 promoter was shortened to not include the full sequence of AT1G31274 situated in 5' of the AGO2 locus. The AGO1 promoter was shortened as complementation was observed previously using a promoter of 790bp (Mallory et al., 2009).

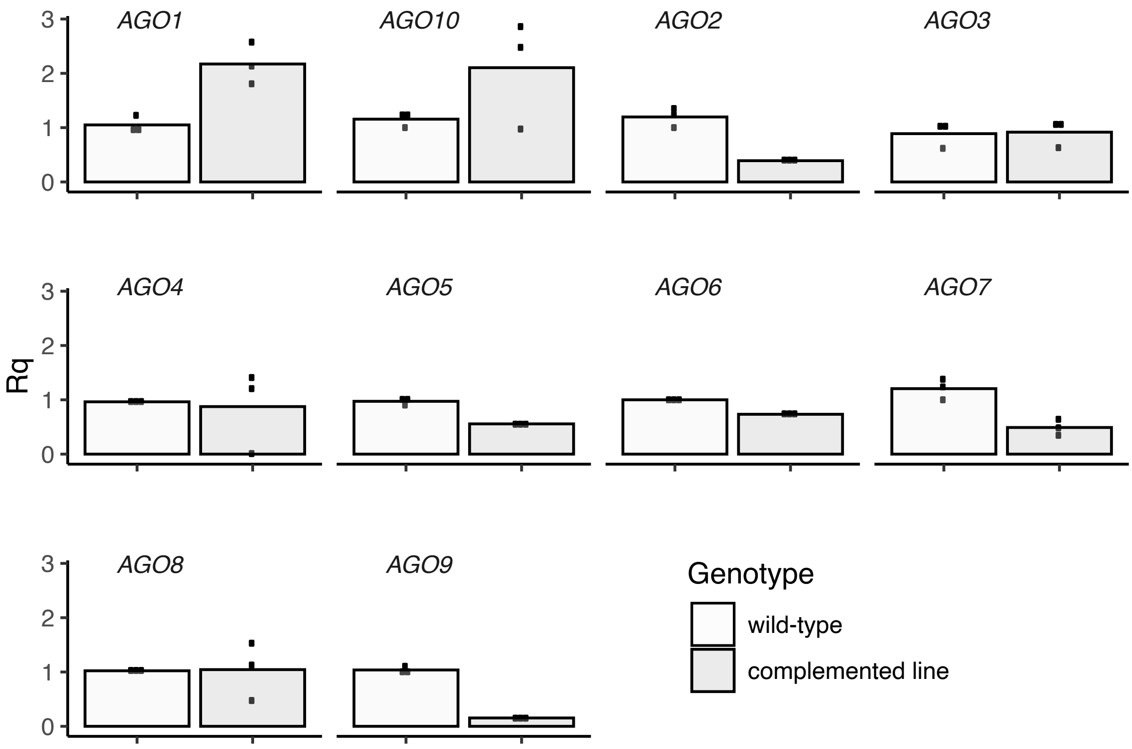

Supplemental Figure S2. RT-qPCR results assessing AGOs expression levels in wild-type and complemented lines. The name of each AGO analyzed is indicated above each graph. ACT2 was used as endogenous control. Three biological replicates of inflorescences are represented by dots and the mean by bars. Rq is the Relative quantification obtained by the ddCt method.

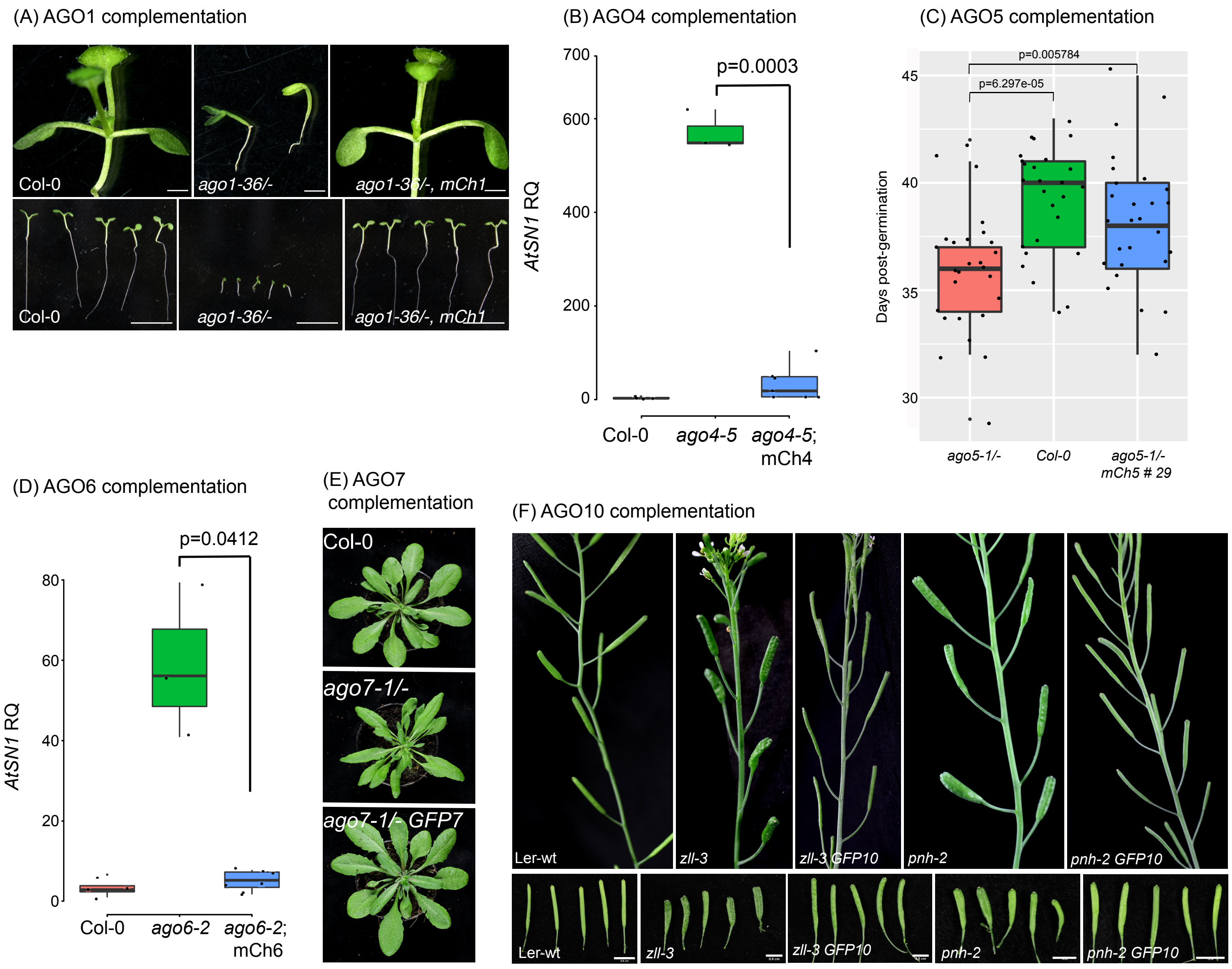

Supplemental Figure S3. Complementation of ago mutants. (A) Complementation of the ago1-36 mutant Arabidopsis by pAGO1:mCherry-AGO1. Representative pictures showing the rescue of the plantlet developmental phenotype in Col-0, ago1-36/- compared to mCh1 #9 ago1-36/-. Scale bars represent 1mm (Top) and 1cm (Bottom). (B) Complementation of ago4-5 mutant by pAGO4:mCherry-AGO4. RT-qPCR showing the absence of AtSN1 ectopic expression in rosette leaves of seven independent lines expressing the construct pAGO4:mCherry-AGO4 (mCh4) in ago4-5 background (n=7) compared to Col-0 (n=3) and ago4-5 (n=3). ACT2 was used as endogenous control. p indicates the p value obtained by a Student`s T-Test. Each dot represents a biological replicate. (C) Complementation of ago5-1 mutant Arabidopsis by pAGO5:mCherry-AGO5. Quantification of ago5-1/- flowering phenotype in ago5-1/- (n=27), Col-0 (n=26) and mCh5#29 ago5-1/- (n=27). p indicates the p value obtained by a Student`s T-Test. Each dot represents a single plant. (D) Complementation of ago6-2 mutant by pAGO6:mCherry-AGO6. RT-qPCR showing the absence of AtSN1 ectopic expression in rosette leaves of seven independent lines expressing the construct pAGO6:mCherry-AGO6 (mCh6) in ago6-2 background (n=7) compared to Col-0 (n=3) and ago6-2 (n=3). ACT2 was used as endogenous control. p indicates the p value obtained by a Student`s T-Test. Each dot represents a biological replicate. (E) Complementation of ago7-1 mutant Arabidopsis by pAGO7:GFP-AGO7. Illustrative pictures of the leaf “zippy” phenotype of ago7-1/- compared to Col-0 and GFP-AGO7 ago7-1/-, n= 7. (F) Complementation of zll-3 and pnh-2 mutants in Arabidopsis by pAGO10:GFP-AGO10 (Col-0 sequences). Representative pictures showing the rescue of the silique’s developmental phenotype in Ler, zll-3, GFP10 zll-3, pnh-2 and GFP10 pnh-2. Scale bar represents 0.5cm. In boxplot, the center line represents the median, the box limits represent the first and third quartile, bars represent the Max and Min (as calculated by the basic boxplot function of ggplot2, all data points are represented.

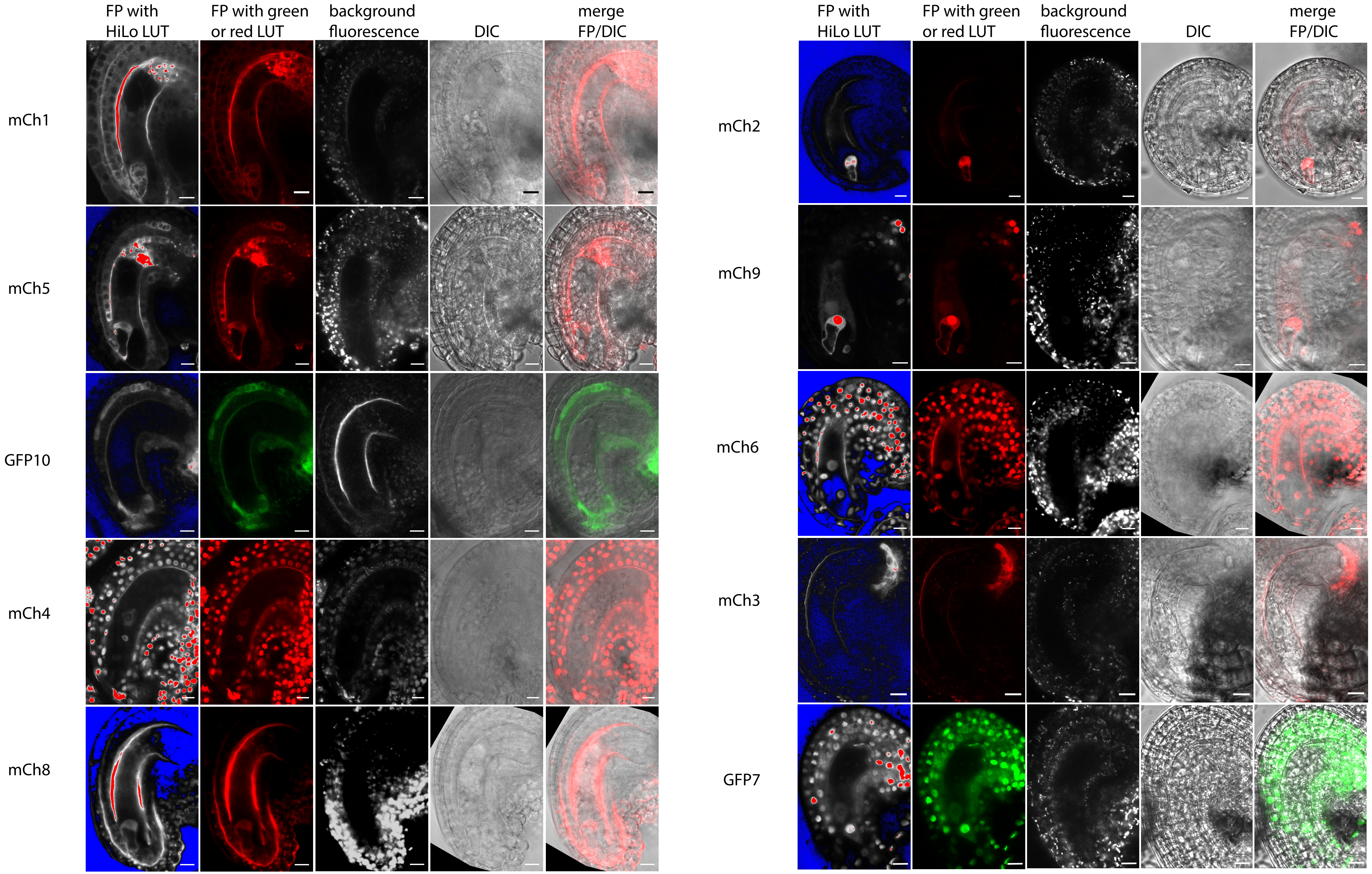

Supplemental Figure S4. Additional channels and LUTs corresponding to the pictures of figure 1. The HiLo LUT represents the images as an intensity grey scale highlighting the under-exposed pixels in blue and the over exposed pixels in red. The green or red LUT show the picture as in figure 1. The background fluorescence represents emissions above ~650nm and correspond to the autofluorescence generated by plastids and/or compounds such as cutin or exine. Abbreviations: Fluorescent protein (FP), lookup table (LUT) and Differential Interference Contrast (DIC). Scale bars represent 10 µm. The name of each AGO line is represented on the left.

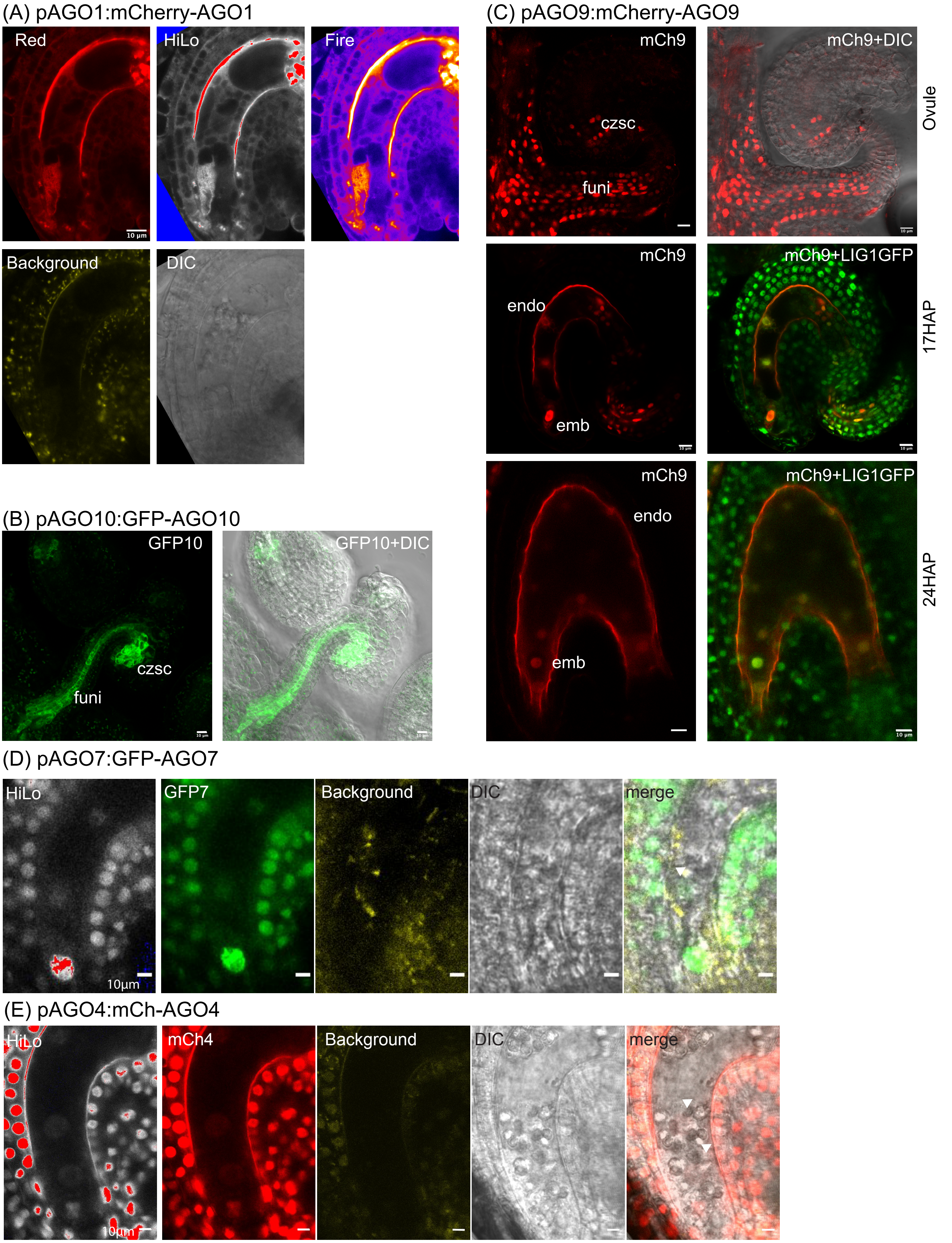

Supplemental Figure S5. Additional pictures (A) Additional picture showing mCh1 expression in the central cell before fertilization. The HiLo LUT represents the image as an intensity grey scale highlighting the under-exposed pixels in blue and the over exposed pixels in red. The fire LUT helps to see the variation in pixel intensity. The background fluorescence represents emissions above ~650nm and correspond to the autofluorescence. Differential Interference Contrast (DIC). Scale bars represent 10 µm. (B) Additional picture showing GFP10 expression in the funiculus (funi) and at the vascular termination of the chalazal seed coat (czsc). Differential Interference Contrast (DIC). Scale bars represent 10 µm. (C) Additional pictures of pAGO9:mCh-AGO9. (Top) Picture of the expression in ovules of mCh9 in the funiculus (funi) and in the chalazal seed coat (czsc). (Middle and Bottom) Accumulation of mCh9 in developing seeds, in early embryo and endosperm, 17 hours after pollination (HAP) (Middle) or 24 HAP (Bottom). Scale bars represent 10 µm. (D-E) Pictures showing GFP7 (D) and mCh4 (E) in early endosperm. Scale bars represent 10 µm.

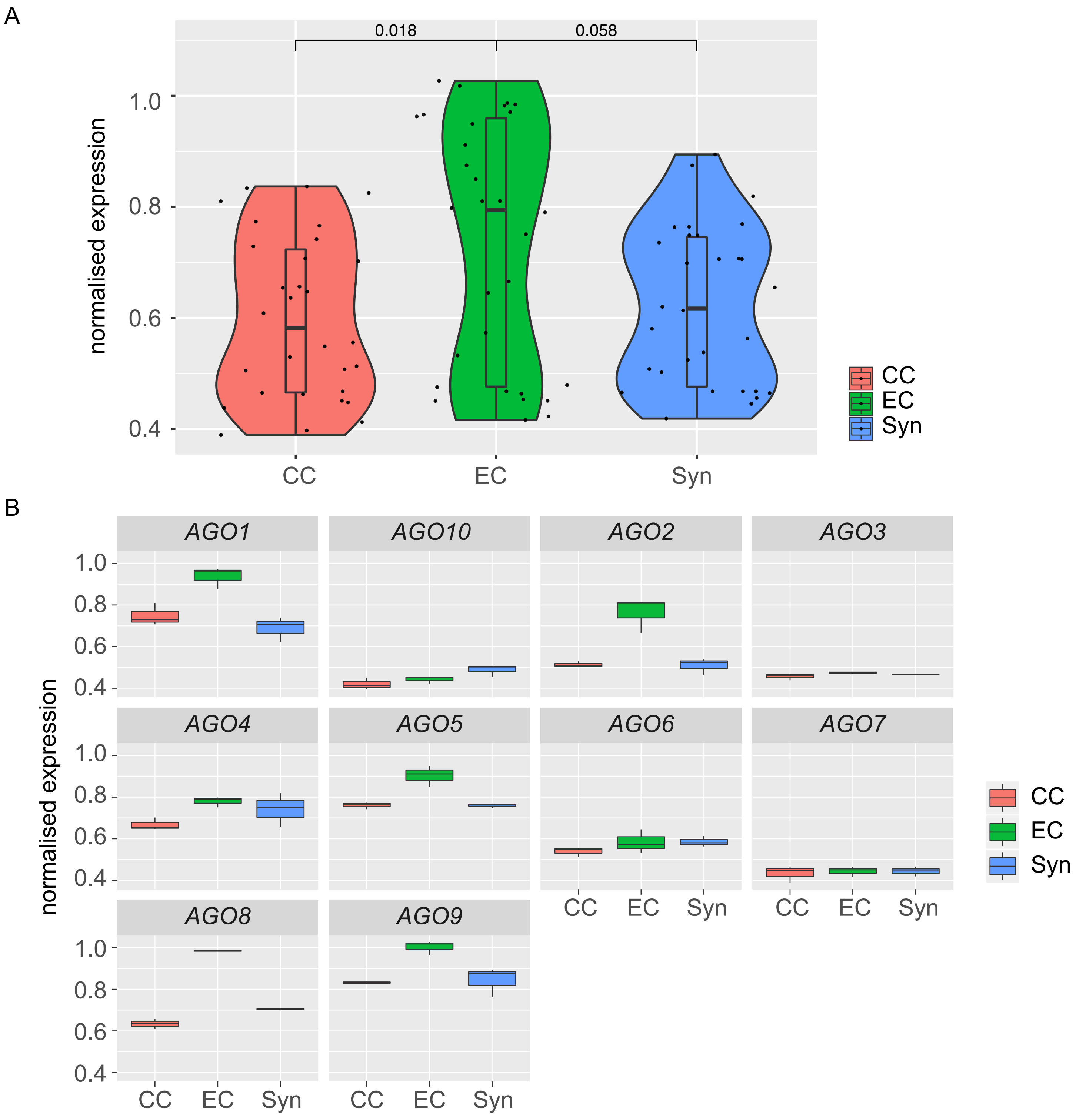

Supplemental Figure S6. Arabidopsis AGO transcription patterns extracted from microarray data of LCM-dissected female gametophytes (Wuest et al, 2010) confirming the general enrichment of AGO transcripts in the egg cell (EC) compared to central cell (CC) or synergids (Syn). (A) Violin plot representing the general enrichment of AGO transcripts in the EC. (B) AGOs individual expression boxplot in the different cell types. p values of a Wilcoxon test are indicated. In boxplot, the center line represents the median, the box limits represent the first and third quartile, bars represent the Max and Min (as calculated by the basic boxplot function of ggplot2, all data points are represented.

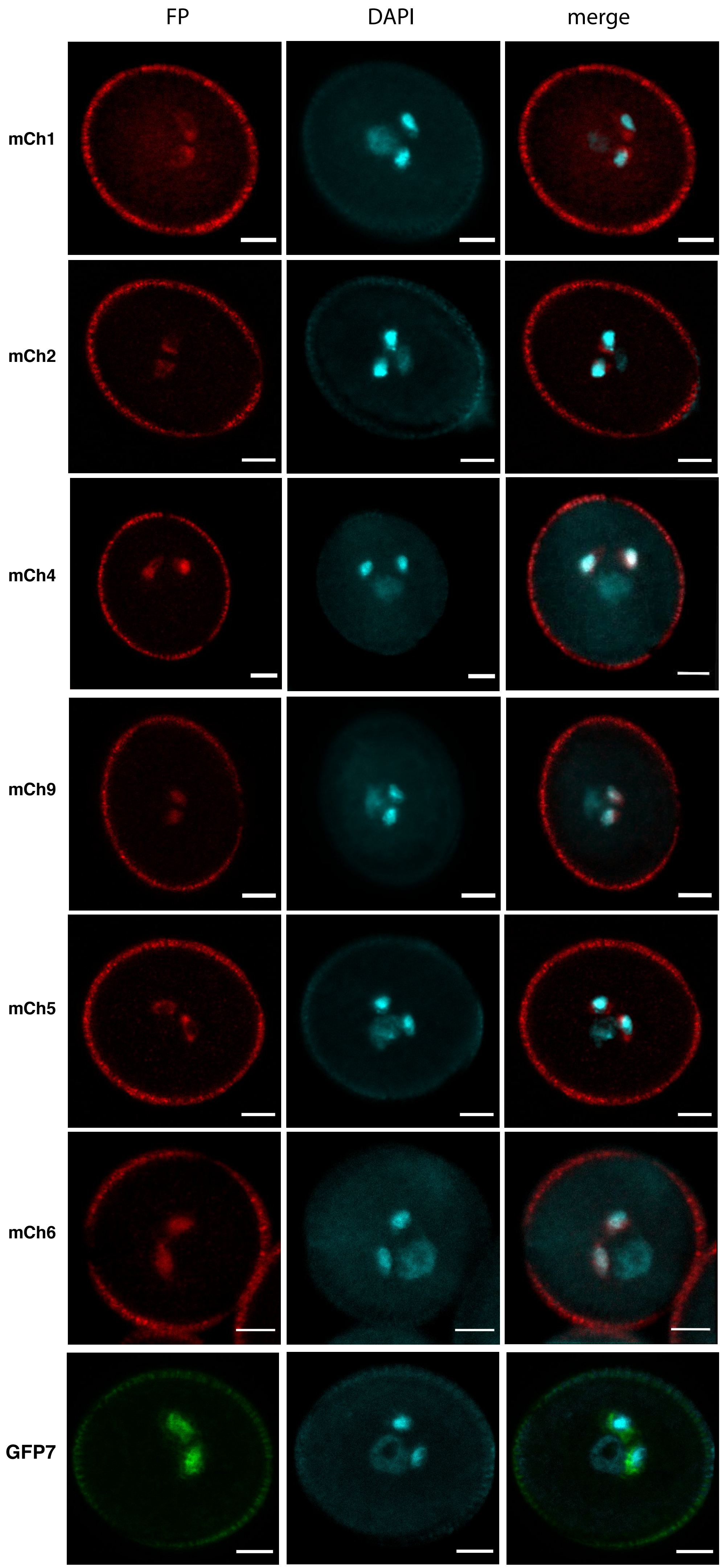

Supplemental Figure S7. Additional pictures of AGOs expression pattern in mature pollen counter stained with DAPI. AGO line name is indicated on the left. Scale bars represent 5 µm.

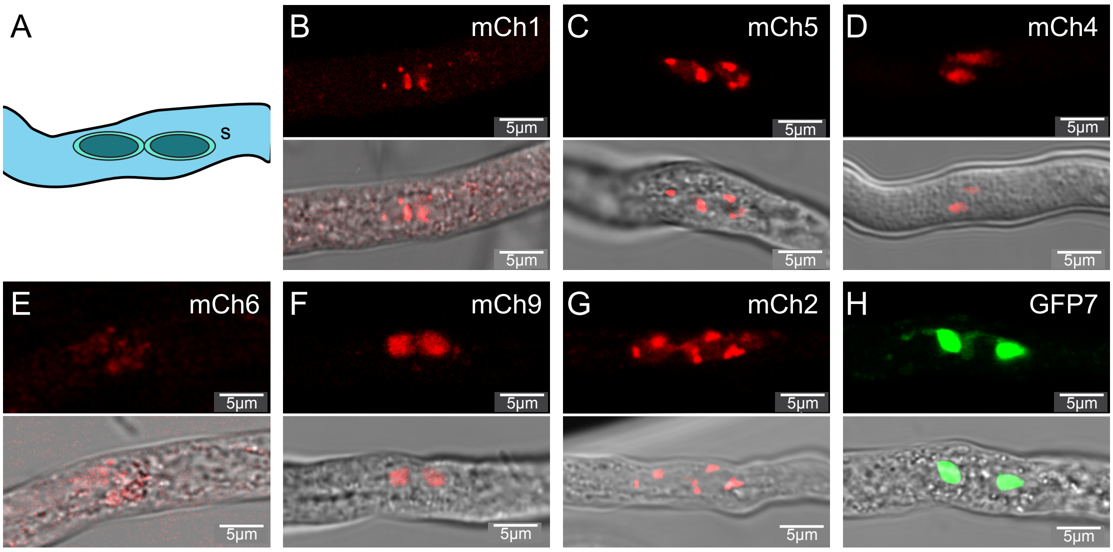

Supplemental Figure S8. AGO accumulation in germinating pollen tube. (A) Schematic representation of a growing pollen tube of Arabidopsis thaliana illustrating the two sperm cells (s). (B-H) Confocal images of the seven Arabidopsis AGOs in germinated pollen grain: mCherry-AGO1 (B), mCherry-AGO5 (C), mCherry-AGO4 (D), mCherry-AGO6 (E), mCherry-AGO9 (F), mCherry-AGO2 (G) and GFP-AGO7 (H). For each AGOs, the upper picture represents the fluorescence alone and the lower picture represents the merge of DIC and fluorescence. Scale bars represent 5 µm.

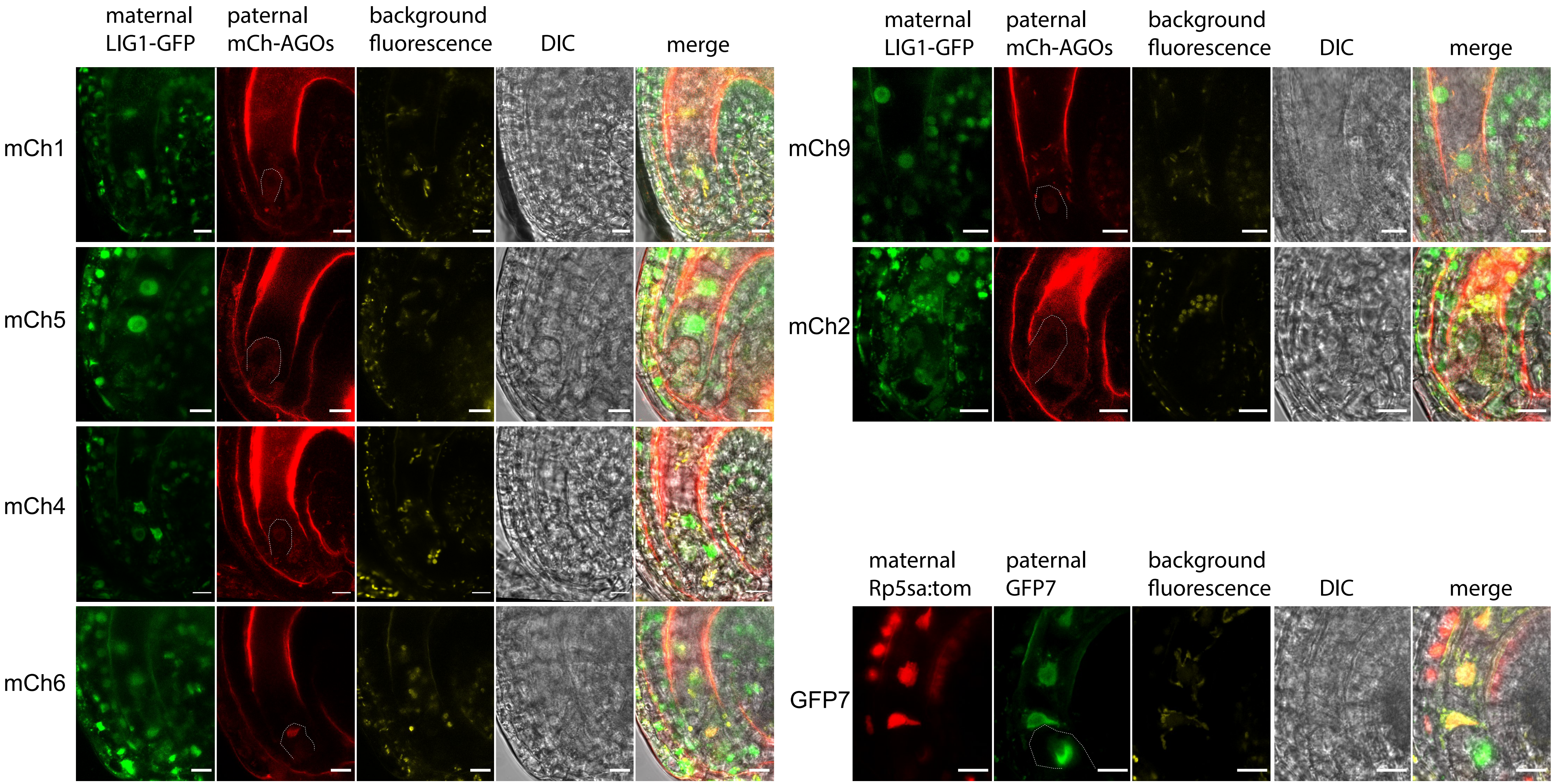

Supplemental Figure S9. Paternal expression in the early zygote of AGOs expressed in sperm cells. Crosses observed at 17 hours after pollination (17 HAP) using LIG1-GFP or RPS5a-tdtomato (nuclear markers) as mother and FP-AGOs as father. Dashed lines highlight the location of the early zygote. We could detect the paternal expression of all analyzed AGO at very low level. Differential Interference Contrast (DIC). Scale bars represent 10 µm. AGO line name is indicated on the left.

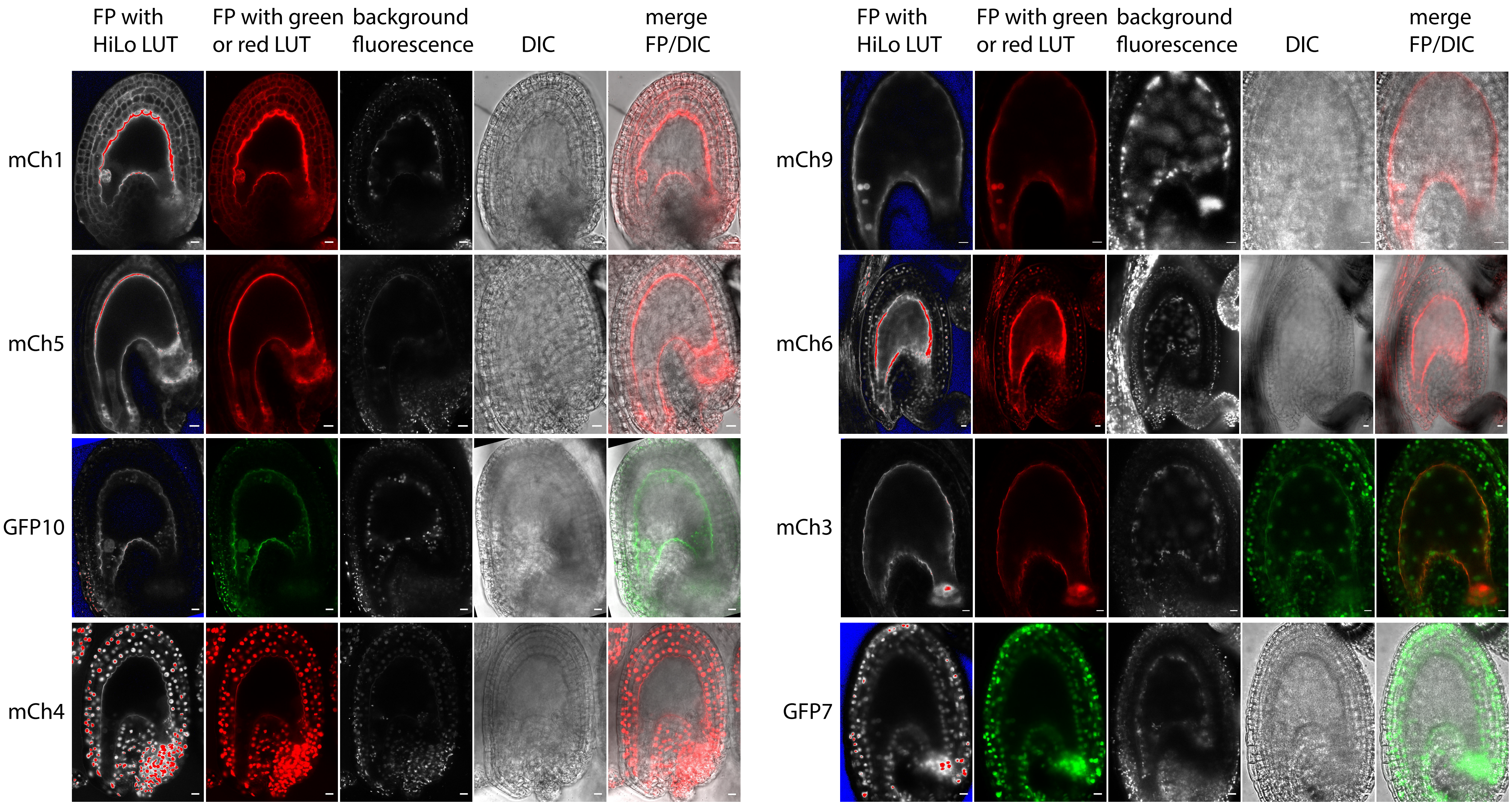

Supplemental Figure S10. Additional channels and LUT corresponding to the pictures of figure 2A-I. The HiLo LUT represents the images as an intensity grey scale highlighting the under-exposed pixels in blue and the over exposed pixels in red. The green or red LUT show the picture as in figure 3A-I. The background fluorescence represents emissions above ~650nm and correspond to the autofluorescence generated by plastids and/or compounds such as cutin or exine. Abbreviations: Fluorescent protein (FP), lookup table (LUT) and Differential Interference Contrast (DIC). Scale bars represent 10µm. AGO line name is indicated on the left.

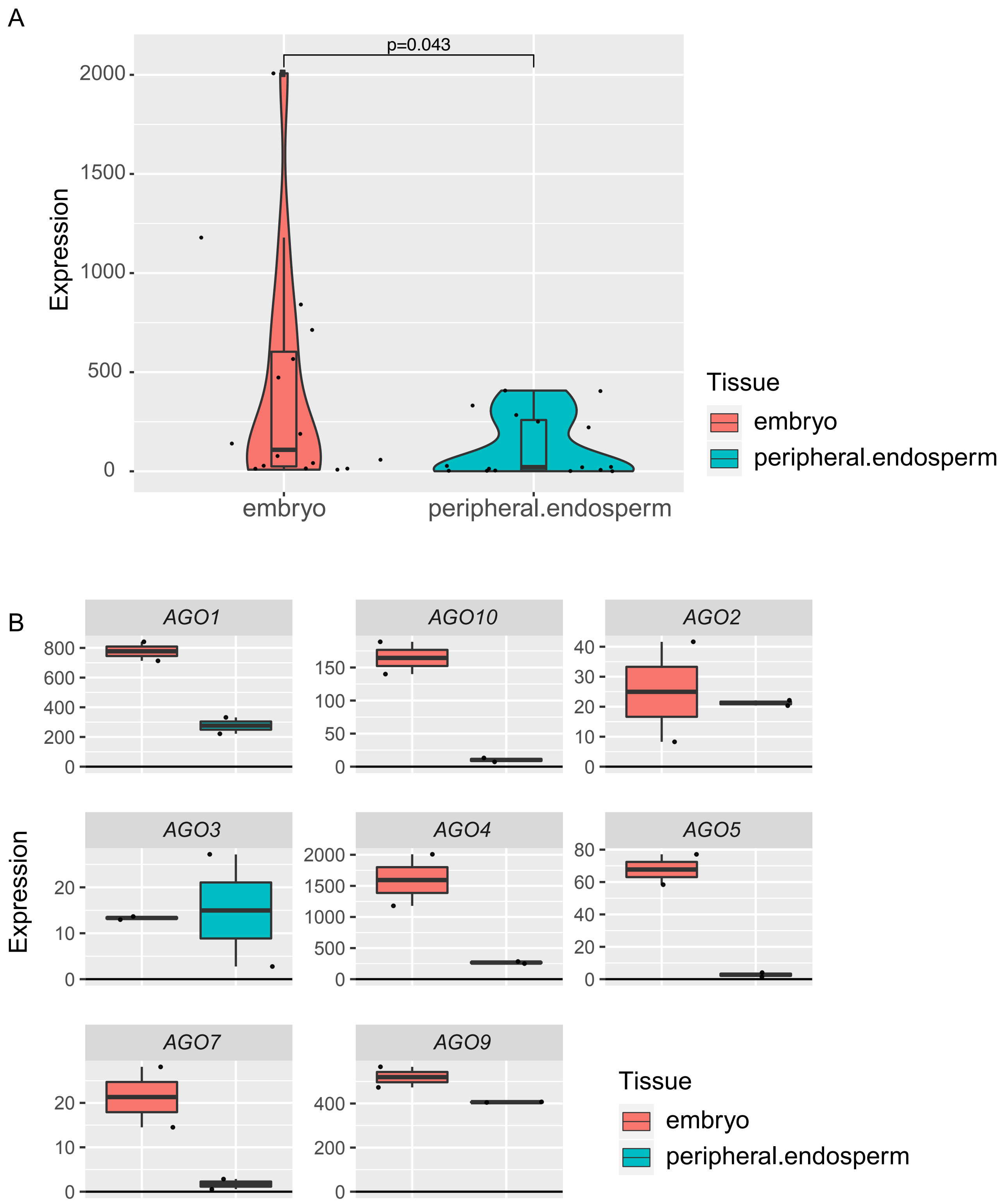

Supplemental Figure S11. Arabidopsis AGO transcription patterns extracted from microarray data of LCM-dissected seeds at the pre-globular stage (Belmonte et al., 2013) confirming the general enrichment of AGO transcripts in the embryo compared to the peripheral endosperm. (A) Violin plot representing the general enrichment of AGO transcripts in the embryo. (B) AGOs individual expression boxplot in the different cell types. p values of a Wilcoxon test are indicated. AGO6 and AGO8 probes are not present in these data. In boxplot, the center line represents the median, the box limits represent the first and third quartile, bars represent the Max and Min (as calculated by the basic boxplot function of ggplot2, all data points are represented.

Supplemental Table S1 . Primers used in this study.

name	sequence	experiment
Ago1CDS_2r-3_F:	GGGGACAGCTTTCTTGTACAAAGTGGGAATGGTGAGAAAGAGAAGAACGGATG	Cloning
Ago1CDS_2r-3_R:	GGGGACAACTTTGTATAATAAAGTTGGGCAAACGCATGAAATCAGTGA	Cloning
pAgo1_4-1r_F:	GGGGACAACTTTGTATAGAAAAGTTGTGACAGCCACCACATTCCTAAAG	Cloning
pAgo1_4-1r_R:	GGGGACTGCTTTTTTGTACAAACTTGATGATTCCTGTGAAAATAACACAACC	Cloning
NP073_pAgo10_4-1r_F	GGGG ACA ACT TTG TAT AGA AAA GTT G atAGGCCGGTGGTTTGCA	Cloning
NP074_pAgo10_4-1r_R	GGGG AC TGC TTT TTT GTA CAA ACT TG tttttgttgtttggattttcaaaaac	Cloning
NP075_Ago10_2r-3_F	GGGG ACA GCT TTC TTG TAC AAA GTG Gga ATGCCGATTAGGCAAATGAA	Cloning
NP076_Ago10_2r-3_R	GGGG AC AAC TTT GTA TAA TAA AGT TG catcccgcgggttaaaatc	Cloning
Ago2CDS_2r-3_F	GGGG ACA GCT TTC TTG TAC AAA GTG Gga ATGGAGAGAGGTGGTTATCGAGGA	Cloning
Ago2CDS_2r-3_R	GGGG AC AAC TTT GTA TAA TAA AGT tg tggtagtccacaaggggca	Cloning
pAgo2_4-1r_F	GGGG ACA ACT TTG TAT AGA AAA GTT G cacaatttggtattgtgcatccg	Cloning
pAgo2_4-1r_R	GGGG AC TGC TTT TTT GTA CAA ACT TG tggatctgatcgggaaacactg	Cloning
PJ-0007-AttB4-pAGO3	ggggacaactttgtatagaaaagttgctcagaacatgactattactgactgac	Cloning
PJ-0017-AttB1r-pAGO3	GGGGACTGCTTTTTTGTACAAACTTGCGGTATTTGACTGGATACGGTCGGGA	Cloning
PJ-0027-AttB2r-AGO3	ggggacagctttcttgtacaaagtgg ct atggatcgaggtggttaccgaggag	Cloning
PJ-0031-AttB3-AGO3	GGGGACAACTTTGTATAATAAAGTTGC TAAAATGTGAATAAGATAGAGTATA	Cloning
PJ-0232-AttB4-pAGO4	GGGGACAACTTTGTATAGAAAAGTTGCTTGTCCGATACTCTCATGT	Cloning
PJ-0233-AttB1r-pAGO4	GGGGACTGCTTTTTTGTACAAACTTGCCTCCTGCTCAAAGAAACC	Cloning
PJ-0234-AttB2r-AGO4	GGGGACAGCTTTCTTGTACAAAGTGG CTATGGATTCAACAAATGGT	Cloning
PJ-0235-AttB3-AGO4	GGGGACAACTTTGTATAATAAAGTTGCAAC TATTAAAGTTTGAGACACAT	Cloning
PJ-0009-AttB4-pAGO5	ggggacaactttgtatagaaaagttgctcacaaatccatttacaaatttaatt	Cloning
PJ-0019-AttB1r-pAGO5	GGGGACTGCTTTTTTGTACAAACTTGCTGTAGCGGAAAGCTTCCCAAAGTAG	Cloning
PJ-0029-AttB2r-AGO5	ggggacagctttcttgtacaaagtgg ctatgtcaaatcgtggtggtggtggtc	Cloning
PJ-0033-AttB3-AGO5	GGGGACAACTTTGTATAATAAAGTTGCAACAAAATGATTAAAGCCAGTGTTT	Cloning
PJ-0317-pAGO6_AttB1r	GGGGACTGCTTTTTTGTACAAACTTGC CCTTCCAGGGGAAAATAATCTGCAA	Cloning
PJ-0318-pAGO6_AttB4	GGGGACAACTTTGTATAGAAAAGTTGCT TGAGAAAGCAAACTTAAAGAGGAAA	Cloning
PJ-0319-AGO6_AttB2r	GGGGACAGCTTTCTTGTACAAAGTGGCT ATGGAGACATCTTCATCTCTGCCAC	Cloning
PJ-0320-AGO6_AttB3	GGGGACAACTTTGTATAATAAAGTTGC TTTAGGAGCTTAAACACGAAGAAGA	Cloning
PJ-0010-AttB4-pAGO8	ggggacaactttgtatagaaaagttgctaaagatctgtttgttgaaagggaat	Cloning
PJ-0020-AttB1r-pAGO8	GGGGACTGCTTTTTTGTACAAACTTGCTTCTCGGCCTCCAGATGTTATGCGT	Cloning
PJ-0030-AttB2r-AGO8	ggggacagctttcttgtacaaagtgg ctatggatacgactctaccgcctcctc	Cloning
PJ-0034-AttB3-AGO8	GGGGACAACTTTGTATAATAAAGTTGCATCGGTTGTGATTCCTTCTTCACCG	Cloning
PJ-0008-AttB4-pAGO9	ggggacaactttgtatagaaaagttgcttttgaggtgatgacaaaaaaaaaat	Cloning
PJ-0018-AttB1r-pAGO9	GGGGACTGCTTTTTTGTACAAACTTGCCTCTGGGATACAAATATCAGAAGGT	Cloning
PJ-0028-AttB2r-AGO9	ggggacagctttcttgtacaaagtgg ctatggattctgatgaaccgaatggga	Cloning
PJ-0032-AttB3-AGO9	GGGGACAACTTTGTATAATAAAGTTGC ACAATTTTATATAGATAGTACATTT	Cloning
GFP 1-2 F	GGGGACAAGTTTGTACAAAAAAGCAGGCTTAATGGTGAGCAAGGGCGAGGA	Cloning
GFP 1-2 R	GGGGACCACTTTGTACAAGAAAGCTGGGTCTCCCTTGTACAGCTCGTCCATGCCG	Cloning
mcherry_1-2_F:	GGGGACAAGTTTGTACAAAAAAGCAGGCTTAATGGTGAGCAAGGGCGAG	Cloning
mcherry_1-2_R:	GGGGACCACTTTGTACAAGAAAGCTGGGTCTCCCTTGTACAGCTCGTCCATGC	Cloning
PJ-0064-AGO1-qPCRR1	GGTCGAGAAGTGCCCTGAAT	qPCR
PJ-0065-MET1-qPCRF1	CGTGGTTCCAAAAGGAGATAAG	qPCR
PJ-0047-AGO10-qPCRF1	GCTTCTTGGAAAGGATGTCTTG	qPCR
PJ-0048-AGO10-qPCRR1	CATTCGCTCTGTGAGTAACCTCTAC	qPCR
PJ-0045-AGO2-qPCRF1	GTAAGCATGGTGGGGCTCGTCCAAC	qPCR
PJ-0046-AGO2-qPCRR1	CCCGGTACTGATCACCTCCTTTAG	qPCR
PJ-0037-AGO3-qPCRF1	TCTTTGACTTGTGCTTCACGTT	qPCR
PJ-0038-AGO3-qPCRR1	TCCATTGTGACAGAAGAGGAT	qPCR
PJ-0041-AGO4-qPCRF1	TGGAACTGAAGACGATGGCATT	qPCR
PJ-0042-AGO4-qPCRR1	TTTTGAGCCACCAACAAAAGGA	qPCR
PJ-0051-AGO5-qPCRF2	GATCCACAACGTGGGCTAGT	qPCR
PJ-0052-AGO5-qPCRR2	GTGTGTGGTGACGTTTCTGG	qPCR
PJ-0043-AGO6-qPCRF1	TCGGGCAGATGTTCCTTCAGTA	qPCR
PJ-0044-AGO6-qPCRR1	GCCCTGCTTGTCCGATAGAACT	qPCR
PJ-0053-AGO7-qPCRF1	TCCTCTTCCTCCTTTACTTCCTCTT	qPCR
PJ-0054-AGO7-qPCRR1	TGCTGCTTTCTCTCCACAACT	qPCR
PJ-0055-AGO8-qPCRF1	TCAAGTCGAAGGCCGCGTGT	qPCR
PJ-0056-AGO8-qPCRR1	TGTGGCTGGCTTGTTCTTGAAGT	qPCR
PJ-0059-AGO9-qPCRF1	TGATGCAGGCATGCAAGTTTCTTGA	qPCR
PJ-0060-AGO9-qPCRR1	ATGCCAGCATGGGCGCAGAG	qPCR
PJ-0650-Act2-qPCRF1	GCACCCTGTTCTTCTTACCG	qPCR
PJ-0651-Act2-qPCRR1	AACCCTCGTAGATTGGCACA	qPCR
PEJ_1092_AtSN1-F	CCAGAAATTCATCTTCTTTGGAAAAG	qPCR
PEJ_1093_AtSN1-R	GCCCAGTGGTAAATCTCTCAGATAGA	qPCR
